# 

**Published:** 1969-10

**Authors:** 


					\
October 1909
IOuAL LIBRARY OF MEDICINE
^cd. on? n o 1969
wov e
.j'V'lJT
DATE: Qc'1 J (Jj
PRIORITY: T~
INCORPORATING THE MEDICAL JOURNAL OF THE SOUTH WEST
84 (/v) No 312
Ki?r ?f'V

				

